# Dimensions of Leader Anger Expression Unveiled: How Anger Intensity and Gender of Leader and Observer Affect Perceptions of Leadership Effectiveness and Status Conferral

**DOI:** 10.3389/fpsyg.2020.01237

**Published:** 2020-07-03

**Authors:** Dongwon Yun, Heajung Jung, Kelly Ashihara

**Affiliations:** School of Business, Konkuk University, Seoul, South Korea

**Keywords:** leader anger expression, leadership effectiveness, status conferral, intensity, gender

## Abstract

While significant organizational research evaluates effective leadership traits and decades of psychological research have investigated emotion, there is a lack of consensus in organizational behavior research related to whether emotion expressed by a leader elicits positive or negative outcomes. We seek to augment existing research by exploring the effect of three dimensions, namely, the intensity of anger expressed, the gender of the leader, and the gender of the observer on perceptions of leadership effectiveness and future status conferral. In Study 1, we recruited 40 participants from a well-known U.S. university to select appropriate terms attributable to intense, moderate, and neutral levels of anger expression. In Study 2, we recruited a diverse pool of 296 participants and employed a quasi-experimental method by randomly assigning participants into one of the six conditions created by three levels of anger expression intensity and the leader gender. Participants were asked to read a vignette in which a male or a female leader responds to an anger-provoking situation with different levels of anger expression and evaluate the leader on perceived leadership effectiveness and future status conferral. Our study findings demonstrated that a leader demonstrating no anger was perceived higher in leadership effectiveness than a leader showing either moderate or intense anger. Juxtaposed to these results, for future status conferral, a leader expressing no anger was perceived as higher in future status than a leader expressing moderate anger without significant difference found between no anger and intense anger. We also found a significant main effect of observer gender with female participants giving lower ratings for leadership effectiveness and status conferral than male participants. Possible explanations and practical implications regarding gender-specific findings are discussed.

## Introduction

On the current world stage, we see authoritative leaders displaying anger as a means to assert power and dominance. While some question the appropriateness of anger and underlying motives, others unwittingly accept and acknowledge such expression as synonymous of power. Recently, U.S. presidential candidate Senator Elizabeth Warren said “I am angry and I own it” and was quoted as saying “We are told that women are not allowed to be angry. It makes us unattractive to powerful men who want us to be quiet.” Likewise, in the 2008 presidential race, Secretary of State Hilary Clinton and Carly Fiorina were criticized for their temperaments. But are these female leaders any more intense than their male counterparts such as Bill Gates, Steve Jobs, Jeff Bezos, or Elon Musk who are known to be visionary geniuses that demonstrate effective leadership and are granted unequivocal status? Though with arguably varying degrees of leadership effectiveness, concurrent political leaders and corporate leaders are known for, among other things, their temperament.

Based on emotional intelligence research (Goleman, 1998), the ability to manage emotions and control impulses is a decisive life skill. Within leadership studies, transformational leaders are known to effectively use emotion to communicate their vision and to motivate followers (Conger and Kanungo, 1987). Likewise, critical leaders with volatile personality are cited as often having a significant negative impact on attitudinal, cognitive, and behavioral performance outcomes in their organizations (Barsade et al., 2018). Consistently, leader disposition and display of emotion are known to considerably impact subordinates who observe and interact with the leader (Yukl, 2005; Titrek et al., 2014). In this context, anger expression by leaders can be known to be emotionally contagious that can set off a process that influences the emotions of others and contributes to organizational norms and culture (Barsade, 2002).

We seek to clarify how leader anger expression in a situational response influences the perceptions of leader effectiveness and conferral of future status. Along these lines, research on leader anger display and leadership perception indicates that leaders who display anger are seen as less effective than leaders who display no anger (Rafaeli and Sutton, 1987; Lewis, 2000) and that the expression of anger is often associated with negative outcomes (Barsade, 2002; Dunn and Schweitzer, 2005; Gino and Schweitzer, 2008). In contrast, considerable research finds that anger display also can be associated with higher status than neutrally tempered leaders (Huy, 1999; Tiedens et al., 2000; Lerner and Tiedens, 2006). Considering contradictory findings related to expression of anger negatively impacting perception of leadership effectiveness but positively conferring status, we are left with another question: in the same hypothetical situation, would leader display of anger in response to a particular situation affect perceptions of leadership effectiveness and conferral of status similarly? Although numerous studies consider either leadership effectiveness or status conferral, few studies compare and contrast whether response to the same situation may evoke similar or contrasting responses in terms of leadership and status. Side-by-side comparison enables us to examine these two aspects of leader perception, which provides clarity related to any similarities or differences in outcomes.

In addressing this question, we first opted to explore more subtle dimensions embedded in anger expression. In previous studies (Tiedens, 2001; Brescoll and Uhlmann, 2008), anger as a high-power emotion is compared to sadness as a low-power emotion. In other research, we see the categorization of expression as either “angry” or “not angry,” a distinction that is rather black-or-white, and this somewhat binary approach neglects one important factor, expression intensity. We ask whether the appropriateness of anger display might be related to the way anger is expressed. How would a leader showing anger intensely be perceived compared to a leader showing anger in a more controlled manner? We believe that consideration of the dimension of intensity will elicit understanding about the effect of leader anger expression on our dependent variables, perceptions of leadership effectiveness, and future status.

Next, we consider the dimension of leader gender and assess whether the gender plays a significant role in findings. Anger is a gendered emotion, and gender studies often find that female leaders who show masculine characteristics such as being assertive, aggressive, and competitive face social sanctions such as being less liked or seen less capable (Watson and Hoffman, 2004; Moss-Racusin and Rudman, 2010; Elsesser and Lever, 2011). Specifying the gender may offer insights into female leadership and organizational behavior as there is still lack of research related to female leadership and understanding of bias that may affect promotion of educated women and difficult-to-discern factors that hinder their attempts to ascend to upper echelons of leadership (Ashihara et al., 2019). In other words, we ask whether a female leader expressing anger is perceived as equally effective and conferred the same unequivocal status as her male equivalent and whether that result varies with the intensity of her anger expression.

Lastly, we examine the gender of observer as a third dimension to consider. Women in high rank are known to suffer from the discrepancy between socially imposed gender norms and managerial expectation of demonstrating rather masculine leadership qualities. In terms of organizational behavior, we think it is fascinating to try to unveil how perceptions of male observers may differ from female observers related to male and female leaders. For instance, are women likely to punish an angry male leader as such display accentuates gender differences and innate physical differences that may instill fear in women? When a female leader responds with anger, which is considered as gender-deviant behavior, are they viewed differently by male and female observers who are subject to their own predisposed views on gender?

In summary, the aim of this research is to explore somewhat paradoxical research findings related to whether leader display of anger similarly affects leadership effectiveness and status conferral. We specified the intensity of expressed emotion and manipulated the degree of anger expression by using preselected terms to create intense, moderate, and neutral anger conditions. In a hypothetical vignette, we then examine how different levels of leader anger expression affect observer perceptions of leadership effectiveness and future status conferral. Next, we considered the possible effect of the leader gender and examined the role of observer gender as men and women are likely to perceive leader behavior differently depending on their innately gendered perspective and embedded norms that may subjectively affect their evaluation.

## Theoretical Development

### Clarifying the Fuzziness of Anger Expression

While emotions such as anger, fear, and sadness are clearly felt by the individual experiencing the emotion, specific definitions related to the facial expressions, vocal expression, and actions are “fuzzy” sets (Russell, 1980; Shaver et al., 1987). While English speakers around the world may easily categorize differences between emotions such as happy, sad, fear, and anger, one step beyond creates a lack of clarity related to how intensity can and should be measured.

Emotions elicit our intuition to take actions (Frijda et al., 1989). For example, while an individual experiencing fear may flee from danger and seek refuge, the sad person may become withdrawn and focus inwardly on their own state of emotion. The angry person, in contrast, becomes stronger and more energized and seeks to move against and rectify the injustice – to reassert their power or status and restore their version of the state of affairs (Shaver et al., 1987; Jung and Young, 2019). In these actions, the person expressing anger may respond in an outraged posture and communicate their anger verbally in a loud voice by yelling, screaming, shouting, and snapping at another party and exhibit non-verbal cues through body expression such as stomping, stalking, or striding. Facially, the angry individual is likely to scowl, glare, stare down, and frown with physical signs of being flush or heated with anger. Shaver et al. (1987) used a prototype model to categorize emotions of fear, sadness, anger, joy, and love.

Prior research on interpersonal effects of emotion primarily focuses on negative emotions (e.g., anger, fear, and anxiety). In particular, anger was one of the most frequently studied emotions (Van Kleef and Côté, 2007; O’Neill et al., 2009; Geddes et al., 2020), as anger is more expressive and easier to observe with concurrent display of distinct and unmistakable expressions such as lowered eyebrows, flared nostrils, and a loud voice (Ekman, 1984). In attempt to integrate a new dimension of intensity, it is first important to clarify term usage categorization for anger expression. The primary objective of our study is to identify terms to differentiate intense, moderate, and neutral expressions of anger.

### Anger Expression in Relation to Leadership Perception and Status Conferral

Leadership is traditionally seen as a distinctly interpersonal phenomenon demonstrated in the interactions between leaders and subordinates. In an organizational context, leadership is often viewed in terms of one’s ability to respond appropriately. Traits of an effective leader include self-confidence, integrity, intelligence, and a sense of humor (Kenny and Zaccaro, 1983) as well as appropriate expression and emotional balance and control (Bass, 1990). One’s ability to manage their own emotions contributes to their ability to handle the needs of employees and effectively motivate them (Cooper and Sawaf, 1997; Goleman, 1998; Ryback, 1998). From a leader’s perspective, organizational constituencies and stakeholders pose significant potential restrictions. At this juncture, we see an intersection between leadership effectiveness and status conferral.

Scholars in sociology, social psychology, and anthropology have long documented that status orderings among individuals and groups emerge naturally in all social contexts (Homans, 1950; Goffman, 1957; Blau, 1964; Frank, 1985; Eagly, 1997; Brewer and Brown, 1998; Chen et al., 2003; Sidanius and Pratto, 2003). Indeed, status is a fundamental determinant of social behavior in interpersonal, intra-group, and inter-group dynamics. Demographic characteristics such as race, age, and gender (Berger et al., 1972) also influence status due to their impact on perceived competence (Li et al., 2016). In business organizations, promotion and compensation confer status; in democracies, votes confer status. Regardless of the context, there is emerging consensus that regardless of whether status is achieved or ascribed, it is voluntarily conferred and, thus, resides in the eyes of those conferring it (Ridgeway and Erickson, 2000; Magee and Galinsky, 2008; Chen et al., 2012). Important to note is that in contrast to leadership effectiveness, status follows from the reality that it may be conferred based on perceived or expected – and not on actual or demonstrated – competence (Fragale, 2006).

While leadership theory addresses effective and ineffective traits and behaviors and status theory looks at the social process of status conferral, such literature typically does not examine the role of emotional expression. In other words, there is often a lack of clarity as to whether the emotional display of anger is viewed positively, thereby considered as trusted and construing benefit to the expressor or viewed negatively, indicating disagreement with the leader’s response and evaluation of anger as inappropriate. Existing research provides somewhat conflicting answers to this question.

First, a significant proportion of research on personal attributes of leaders explains some variance as to whether a person emerges as a leader and acquires the necessary skills to be effective (Kirkpatick and Locke, 1991). In the role of CEO, anger can be associated with effective leadership when expression is either linked with promise and reward for good performance or threat and discipline for poor performance (Bass, 1990). Anger, even strong anger, can be defined in terms of consequences of goal-directed behaviors and may be warranted when output is critical; a deadline exists; resources are limited; or ethical or moral values are at stake (Keltner and Haidt, 1999). Likewise, anger may have a distinct role in supervisor–subordinate relations. In fulfilling their oversight responsibilities, supervisors may be motivated to display anger to express disapproval of subordinate performance or direct attention to the importance of a task. When followers observe and experience emotional expression of a leader, they may cognitively process and, by empathizing, also come to mimic the emotion (Hatfield et al., 1994; Lewis, 2000). The process of emotional influence is known as emotional contagion (Schoenewolf, 1990; Van Kleef et al., 2010; Schwarzmüller et al., 2018).

In the context of leadership and organizational behavior, anger expression can have a significant negative impact on a variety of attitudinal, cognitive, and behavioral outcomes (Barsade et al., 2018). Anger has been associated with increased tendencies for aggression (Neuman and Baron, 1998) and with performance outcomes such as a leader’s failure to establish strong relationships and sense of trust with team members (Jones and George, 1998; Aquino et al., 2001; Tripp, 2001), decreased group productivity (Jehn, 1995), and lower individual and group task/job performance (Van Kleef et al., 2010). If connected to poor judgment, anger expression may also be viewed as acting outside of leader role norms (Rafaeli and Sutton, 1987), which leads to lower ratings of effectiveness than a neutrally tempered leader (Glomb and Hulin, 1997; Lewis, 2000).

Second, while perception of leadership effectiveness is connected to evaluation of a situation and judgment related to the appropriateness of the leader response to the evoking circumstance, status conferral is linked to inferences of future status with psychological processing relating to diagnostic inference. In a study on the relationship between anger expression and status conferral, Tiedens et al. (2000) hypothesized that the way in which a person communicates and expresses emotion confers social status. In their study, they found that when people read a description of two individuals feeling angry or sad/guilty in the presence of a negative event, they inferred the angry person to be of higher status than the sad or guilty person. In addition to inferring status, people also accorded status in rank and monetary reward according to the emotions displayed. In an experiment, MBA students who played a role of job interviewer accorded more power/status and higher salary to the interviewee who reported anger about a negative event than one who reported sadness or guilt. Moreover, situational studies indicate that display of anger can signal competency and serve as a means of influence to gain legitimacy from others and authority through validity of reasoning (Shields, 2002, 2005). As organizations are often competitive environments with individual employees vying for airtime and recognition, expressing anger can be a means to gain authenticity and, if evaluated as appropriate in degree and expression, can confer benefit and demonstrate authority associated with leadership and promotion.

By connecting these two dependent variables with anger expression in the same hypothetical situation, we seek to clarify whether there are indeed paradoxical findings related to whether a leader showing anger is negatively perceived in terms of leadership effectiveness but positively benefits in terms of status conferral.

### Dimension 1: Anger Expression Intensity

Significant research at the cross section of leadership and emotion display in general and anger in particular looks at anger display as both binary – anger or no anger or in comparison to other emotions such as sadness which is categorized as more feminine interpersonal emotions (Kelly and Hutson-Comeaux, 1999, 2000). In response to this conundrum, rather than comparing two different emotions such as sadness and anger or comparing anger with no anger, we posit that it may be worthwhile to exclusively focus on anger and look at whether inconsistent findings from studies on leadership perception and status conferral be attributed to the negligence of intensity.

For instance, Tiedens (2000) study found that when people read a description of two individuals feeling angry or sad/guilty in the presence of a negative event, they inferred the angry person to be of higher status. Other studies use one anger expression condition which is conveyed through voice tone, facial expression, demeanor, and gesture. In other words, one anger intensity level is compared with a neutral expression condition (Glomb and Hulin, 1997; Tiedens et al., 2000). In this setting, while there is a combination of cues that confirm the anger intensity level, neither the degree of the anger expressed nor the degree to which perception related to anger were measured. If observers perceived the expressed anger to be too intense, their preference for neutral expression could stem from their perception that anger expression was inappropriate in intensity rather than the expression of anger in and of itself.

Our emphasis on the intensity factor is supported by the “Dual-Threshold Model of Anger in Organizations” proposed by Geddes and Callister (2007). In this model, the authors argued that anger expression can be divided into three categories of suppressed anger, expressed anger, and deviant anger according to whether it crosses either the expression threshold or impropriety threshold set up by organizational norms. In other words, when anger is not displayed at all, it falls into suppressed anger category. Once anger is displayed, the expression threshold is crossed. At that time, should there be violation of implicit standards for acceptable expression, the anger becomes “deviant anger” and it crosses the impropriety threshold; otherwise, it remains categorized as “expressed anger.” Geddes and Callister (2007) maintain that suppressed and deviant anger lead to more negative outcomes, while expressed anger is more likely to lead to positive outcomes. Suppressed anger can be destructive in that it prevents existing anger-provoking problems from being communicated and addressed. Deviant anger is detrimental to the organizational harmony in that it violates the social norm around anger expression by infusing hostility and anxiety into the organizational atmosphere. However, anger expressed within the zone between the two thresholds is referred to as in the “zone of expressive tolerance” and seen as accepted by observers and appropriate and value added in the problem-solving process. Since the outcomes discussed by Geddes and Callister (2007) are macro-level and more related to group problem solving than individual gains, we undertook a study to fill a void in empirical research by exploring how expression of anger in different intensity levels impacts perceived leadership effectiveness and status conferral.

Hypothesis 1: Perceptions of leadership effectiveness will differ according to the intensity of the leader’s anger expression.

Hypothesis 2: Future status conferral will differ according to the intensity of the leader’s anger expression.

### Dimension 2: Leader Gender

One of the distinctive characteristics of humans as beings is the capacity to be self-reflective and aware. As emotional beings, our response to a given situation can be judged as either functional or dysfunctional according to the circumstance. Related thereto is the question of perception. Does a leader’s expression of emotion conform to social norms, and as such, is the expression perceived as an appropriate response proportional to the evoking circumstance? Or is the leader’s expressed emotion contrary to social norms and perceived as an inappropriate emotional response or reaction to the situation?

Research related to topics of social appropriateness and display rules often focuses on tacit social rules directing the timing, degree, and way in which emotions should be expressed, emphasizing the importance of regulating emotional expression as a proxy for positive social interaction outcomes (Saarni, 1999; Diefendorff et al., 2005). Emotional display rules refer to the regulation of expressing appropriate emotions in the workplace (Ekman, 1973). Research indicates that leaders whose behavior and attitudes match leadership prototypes are perceived more positively than leaders whose behaviors and attitudes do not (Lord et al., 1984). The degree of appropriateness is linked to the idea that gender stereotypes are composed of traits that are high and low in social desirability with (Prentice and Carranza, 2002) characteristics such as “happy” and “sad” categorized as more feminine interpersonal emotions and anger as more masculine and with lower general desirability (Kelly and Hutson-Comeaux, 1999, 2000).

A fundamental question is whether gender norms of behavior affect whether the expression of a given emotion is seen as an appropriate or inappropriate response. Reconciliation of this question is fundamental to evaluation of emotional expression and, in turn, is related to the external evaluation of the leader and whether, in the case of our hypotheses, anger display is perceived as effective leadership and confers future status. The interpersonal aspect of emotion (Shields, 2002) is related to whether an individual is able to manage and control their emotion and thereby perceived as rational and self-controlled, which in turn increases the likelihood that the individual will be trusted. If categorized as “expressed anger” or within the zone of expressive tolerance, anger is more likely to be perceived as an appropriate in response and hence more authentic leadership. How others view the behavior and their perception as to whether the expressed emotion is legitimate can influence their conferral of future status and perceptions of leadership.

According to the social role theory (Eagly et al., 2000), female managers suffer from the gap between *gender* stereotypes and *managerial* expectations. While gender norms indicate that it is desirable for women to be sensitive and caring, those two qualities are less relevant with good leadership skills (Eagly and Carli, 2003). A leader is expected to be strong, result-oriented, and willing to take risks (Stoker et al., 2012). Thus, female leaders experience the incongruity between gender norms and expectations as a manager (Stuhlmacher and Poitras, 2010), accordingly given evidence of both descriptive and prescriptive biases associated with leader gender, for female leaders to be perceived as effective they should demonstrate both sensitivity and strength while male leaders only needed to demonstrate strength (Johnson et al., 2008).

In recent studies, since anger and pride are emotions that men express more than women (Plant et al., 2000), the origin of the anger expressed by women is seen as more internal (e.g., “she is an angry person” and “she is out of control”) than external (e.g., “the situation is frustrating”). Thus, according to Brescoll and Uhlmann (2008), for the same situation, the male anger display was viewed as a response to objective, external circumstances and female anger display more a product of her personality, which helps explain lower status conferral. As a result, anger display by a female professional may imply she is less competent. These results imply a bias and support the general principle that under the same objective circumstance, gender-incongruent leaders are seen less effective and awarded lower status than gender-congruent leaders.

This connects with our research question: how a female leader who displays anger, a masculine emotion, and acts outside of gendered emotional norms is perceived vs. a male leader who expresses the same emotion and is more in line with gendered emotion norms. In tandem is the quest to understand how the intensity of anger expressed interacts with gender norms. This brings us back to our research topic of how intensity of anger expressed and gender of expressor affect perception of leadership and status conferral and whether female leaders who display anger and act outside of gendered norms are effectively punished with lower leadership evaluations. In other words, is intense anger construed differently for male leaders and female leaders?

Hypothesis 3: The effect of leader anger expression intensity on *perceived leadership effectiveness* will differ according to the gender of the leader.

Hypothesis 4: The effect of leader anger expression intensity on *future status conferral* will differ according to the gender of the leader.

### Dimension 3: Observer Gender

In addition to evaluating the leader gender effect, given that men and women are often described as innately different on the DNA level and the compounding factors of socialization from micro-units of the family to macro levels of society, the reality is that while over 50 percent of the workforce is women, men hold almost two-thirds of management positions, and as of December 2019, women leaders held CEO positions in only 5.8% Fortune 500 companies (Catalyst, 2020). There may be more women in the corner office today than a decade ago, but the gender gap is still very real at the top of the corporate ladder. In attempt to try to identify some factors, we consider the dimension of the observer gender and how it interacts with anger intensity, leader gender, and perceptions of leadership and status. After all, since ultimately women are in real life employees aspiring for leadership positions, how women evaluate male and female leaders may be indicative of the psychological inhibitions they themselves feel and that may later affect their choice to stay the course and “lean in” (Sandberg, 2013) or opt out of the workforce.

In seeking to ascend, women should embrace masculine competencies in order to be perceived as qualified for high-status roles such as leaders; on the other hand, when they do so they risk penalties. In other words, perceivers receive psychological rewards for preserving display rules. Accordingly, there are consequences for gender deviants (Bartol and Butterfield, 1976; Derlega and Chaikin, 1976) who step outside of gender norms (Cialdini and Trost, 1998). Such implies that observers would penalize female atypical behavior (Eagly et al., 1992). While a recent McKinsey study indicated that the stressful demands of work lead women to feel isolated and feel like an “only” as such, and only are more likely to have their ideas challenged, be subject to demeaning remarks, and be sexually harassed resulting in above average exit rates (Kirvkovich et al., 2018), expression of anger may likely exacerbate that already acute sense of isolation and lead women to act in line with in-group norms (Ashihara et al., 2019). In other words, violation of gender norms or gender deviance prevents men and women from expressing their full human capacities without incurring social and economic costs (Rudman and Fairchild, 2004).

To be more specific, we can view the effect of observer gender on their perception of the leader from two different perspectives which are the characteristics of observer gender itself and the match between observer gender and leader gender. It was found that women can be more susceptible and more easily affected by interpersonal mistreatment at workplace than men (Montgomery et al., 2004; Escartin et al., 2011), and this finding indicates that female observers would be more affected by a leader displaying strong anger and likely to evaluate the leader more negatively than male observers. On the other hand, partly based on the similarity/attribution theory by Byrne (1971), since people are more attracted to and tend to develop a more favorable attitude toward individuals who are similar to themselves, there may be a tendency to accord favorable evaluation to a leader of the same gender.

In summary, while we examine whether a female leader is perceived as more effective when she stays calm than when she expresses anger, we also wonder whether women who express anger will be viewed similarly by male and female observers. As Senator Elizabeth Warren posited, do men punish powerful women who are angry, or, aligning with Kelley (1967, 1973), do women observers punish angry women leaders as behaviors are different from gender norms? An equally interesting and relevant question is whether women evaluate angry male leaders significantly lower than male observers as the physical power may create aversion for display of intimidating emotion that accentuates innate gender differences.

Hypothesis 5: The effect of anger expression intensity and leader gender on *perceived leadership effectiveness* will differ according to the gender of the observer.

Hypothesis 6: The effect of anger expression intensity and leader gender on *future status conferral* will differ according to the gender of the observer.

## Research Overview

To address how intensity of anger expressed and gender of leader affect perception of leadership and status conferral, we used a vignette method to expose participants to a situation where anger is expressed (or not) and asked participants to evaluate leadership effectiveness and status conferral of the leader. While in reality the intensity of anger expression is highly variable, we used a hypothetical situation in an attempt to isolate and quantify the effects of our independent variables – anger expressed intense, moderate, or not at all and gender of the leader and gender of the observer.

Study 1 is a preliminary study which intends to find the most appropriate and representative words for describing three intensity levels of anger expression. In Study 1, we listed terms in categories as they related to general emotions, facial emotions, vocal expressions, and physical expressions. Study 2 investigates participant responses related to leadership perception and status conferral according to different levels of anger expression intensity as described by words selected in Study 1 and how the leader gender and observer gender may affect the perceptions of leadership effectiveness and status conferral differently. In addition to adding depth to the binary use of anger and no anger, this study seeks to bridge studies in psychology and sociology with organizational behavior in terms of how affective stimuli such as anger may lead to outcomes of attitude or are related to behavior and performance.

### Study 1: Selection of Terms Expressing Levels of Anger Intensity

Study 1 builds upon the prototype approach and category system of Shaver et al. (1987) to select generic emotional terms, facial expression terms, vocal expression terms, and physical expression terms. To clarify “fuzziness” in term meaning and best select the terms used in the main study related to the specific categories of expression, we first collected terms describing anger experience and expression. We then proceeded to select terms to describe three different intensity levels of anger expression – intense, moderate, and no anger. To compensate for some of the limitations innate to a hypothetical vignette, we included four categories with a total of twenty-seven terms (see Table 1). The breadth of terms allowed us to provide more complete and realistic descriptions of an individual expressing emotion. We then tested terms to determine the appropriateness of intensity level. In narrowing down the selection, we considered means, variance, and range as well as other elements such as familiarity and clarity. The extent to which they were correlated with anger expression is in line with English dictionary definitions and according to common usage of the vocabulary tested.

**TABLE 1:** Descriptive statistics for anger intensity terms by category (Study 1).

Expression Category	Anger terms	Mean	Rank by mean	Variance	Min	Max	Selection category
Generic emotional terms	Outraged	9.78	1	1.69	4	11	
	**Furious**	9.68	2	1.35	6	11	Intense term
	Infuriated	9.05	3	2.31	1	11	
	Rageful	8.75	4	1.9	4	11	
	Irate	7.78	5	2.34	2	11	
	Angry	7.68	6	1.77	3	11	
	Mad	7.08	7	2.08	1	11	
	Resentful	6.35	8	2.57	2	11	
	Irritated	5.98	9	2.01	3	11	
	**Annoyed**	5.6	10	1.91	2	9	Moderate term
	Perturbed	5.33	11	2.52	1	11	
	Grouchy	5.18	12	2.09	2	9	

Facial expression terms	**Glare**	7.48	1	2.44	1	11	Intense term

	Look angrily at	7.28	2	2.45	2	11	
	Scowl	7.03	3	2.42	1	11	
	Glower	6.88	4	2.5	1	11	
	Stare down	6.75	5	2.77	1	11	
	Grimace	6.1	6	2.8	1	11	
	**Frown**	4.49	7	2.15	1	10	Moderate term

Vocal expression terms	**Yell**	8.6	1	2.34	3	11	Intense term

	Scream	8.53	2	2.24	3	11	
	Shout	8.15	3	2.28	4	11	
	**Snap at**	7.44	4	2.6	2	11	Moderate term
	Screech	6.43	5	3.04	1	11	

Physical expression terms	**Stomp**	7.93	1	2.48	2	11	Intense term

	**Stalk**	6.13	2	2.29	2	11	Moderate term
	Stride	3.43	3	2.22	1	9	

#### Participants and Procedure

Forty students and staff (15 males, 25 females) in a well-known public U.S. university on the west coast participated in this study as part of an experiment session run by a behavioral lab in the school of management. Students were recruited from a behavioral lab pool that consists of voluntary participants in behavioral studies and were requested to come to the lab at their scheduled time and participate in several studies. At the end of the session, participants were given a short questionnaire asking them to rate various emotional terms based on the anger intensity conveyed by each term. Upon successful completion of the study, participants were compensated with $3 credit on their school account for use on campus. Mean age was 21.63 years (*SD* = 5.13, *Min*_*age*_ = 18, *Max*_*age*_ = 43).

#### Methods

To evaluate the appropriate selection of anger terms, all candidate vocabulary terms were reviewed and evaluated by outside raters on several dimensions such as frequency of use, familiarity, clarity, and relevance to anger. In total, 27 anger relevant terms were included in the questionnaire. The terms were divided into four term categories: generic emotion, facial expression, vocal expression, and physical expression.

As illustrated in Table 1: *generic emotional* terms category included twelve anger terms: annoyed, furious, outraged, resentful, angry, irritated, mad, infuriated, grouchy, rageful, perturbed, and irate. Related to terms describing *facial expression*, we assessed anger intensity for seven terms: frown, scowl, glare, glower, look angrily at, grimace, and stare down. *Vocal expression* terms category included four terms: yell, scream, snap at, shout, and screech. Related to *physical expression* terms, we tested terms stomp, stalk, and stride. Participants evaluated each term on an eleven-point Likert scale and were asked to rate how much anger the term conveys with “1” indicating no anger and “11” extreme anger.

#### Results and Discussion

The means, variances, and maximum/minimum values of intensity ratings for each anger term are summarized in Table 1. Based on mean values, anger terms showing relatively higher intensity ratings within the same category were classified as candidate words for expression of intense anger, while terms showing lower anger intensity ratings were classified as candidate words for moderate anger expression.

Under the generic emotional term category, “furious” was chosen as the term for intense anger. Furious ranked second by mean, had the lowest variance, and the highest minimum rating (6). Study 1 results are in line with Shaver et al., which on two-dimensional coordinates placed furious as 6th out of twenty-nine, similar in intensity to terms of outrage, hostility, and spite and slightly lower than exasperation and anger. In the facial expression term category, we selected “glare” as the intense term is it ranked first. We opted to include facial expressions related to eye contact as eyes relay emotion. For vocal expression terms, we selected “yell” as it ranked first in intensity. Under the physical expression category, we selected “stomp” as our intense term.

For moderate terms, annoyed was selected as our moderate term as it ranked 10th out of 12 terms with relatively low variance of 1.91. Corroborating our findings, Shaver et al. (1987) ranked annoyance as seventeen out of twenty-nine terms, similar in intensity to irritation and aggravation and slightly less intense than scorn and disgust. “Frown” was selected as our moderate facial expression ranking last as an anger term. Shaver et al. also gave “frowning not smiling” a coding reliability of 1.0. For vocal expression terms, we selected “yell” as it ranked first in intensity and “snap at” was selected which ranked 4th. For physical expression, we selected “stride” as our moderate term.

As a result, one anger expression word for each category was finally chosen as the representative term for intense and moderate anger expression as shown in Table 1. For the intense anger expression condition, furious (generic emotional term), glare at (facial expression term), yell at (vocal expression term), and stomp (bodily expression term) were chosen. For moderate anger expression condition, annoyed (generic emotional term), frown (facial expression term), snap at (vocal expression term), and stride (bodily expression term) were chosen. In the study of organizational behavior, we seek to clarify fuzziness in terminology and add the dimension of intensity to binary studies that only compare anger vs. no anger.

### Study 2: Effects of Anger Expression Intensity and Leader/Observer Gender on Perception of Leadership Effectiveness and Future Status Conferral

This study explores how leader anger expression influences the perception of leadership and status conferral. Participants read a vignette describing an anger-provoking episode between a leader and their assistant and provided their perception of the leader in terms of leadership effectiveness and future status.

To manipulate anger expression intensity, three versions of vignette were created using anger terms expressing intense, moderate, and neutral levels of anger in accordance with Study 1. We intend to investigate whether intensity and gender of leader and observer affect leadership effectiveness and status conferral similarly and how results parallel similar studies by Lewis (2000); Lerner and Tiedens (2006), and Brescoll and Uhlmann (2008). To test for any potential gender effect, two vignette versions were introduced with the only difference being whether the depicted leader was either male or female. Gender differences were integrated as former studies yielded conflicting results depending on the gender of the leader. For instance, according to Glomb and Hulin (1997), angry female supervisors received higher ratings than angry male supervisors, whereas according to Lewis (2000), female leaders were evaluated to be less effective when displaying anger compared to male leaders. On the other hand, in the study by Tiedens (2000), the characters in the vignette were named as X and Y without any gender information provided, so gender effect was not integrated. Since women are usually expected to show less anger than men due to their relationship-oriented tendency (Timmers et al., 1998), since they are subject to social display rules that tacitly direct their expression (Saarni, 1999; Diefendorff et al., 2005), and since anger is seen as a more masculine emotion (Prentice and Carranza, 2002), we predict that the effects of anger expression intensity on leadership perception and status conferral can differ according to the gender of the leader.

We also explored the effect of observer gender to understand how gender and one’s own perceptions may impact their evaluation of leader anger expression on the dependent variables, leadership effectiveness and status conferral. Brescoll and Uhlmann’s (2008) study indicated that female participants rated angry leaders overall much lower than their male counterparts with a much less pronounced difference for leaders expressing no anger. Similarly, Kelley’s attribution model (Kelley, 1967, 1973) connects display rules with anger expression and linking anger expressed by female leaders with internal causes, which contribute to lower conferred status. As a result, professional women may benefit from being “unemotional” so that they are seen as rational (Albright, 2003). Similarly, Lewis (2000) indicates significant differences between the way men and women experience and express emotion and women leaders often evaluated as less effective when exhibiting more masculine styles (Eagly et al., 1992).

#### Participants and Procedure

Study 2 included 311 participants recruited via two different routes. One-hundred-eleven undergraduate students and staff from a well-known public US university were recruited from the same management behavioral lab constituency as in Study 1 and received an e-mail link inviting them to the online study. Participants were recruited and compensated with the cash equivalence of $3 on their school account for use on campus upon completion of the study as instructed. Since emotional terms may have generational context and situational interpretation can vary according to experience in the workplace and relative rank, we recruited two hundred demographically diverse participants via Amazon MTurk and paid $0.50 upon completion of the survey. Non-native English speakers were precluded from participation to ensure proper understanding of the scenario and intended interpretation of the test terms. Among the respondents, twelve people who failed to pass the attention filter and three people who provided outlier responses were excluded, with 296 included in our study analysis. The average age of participants was 27.28 years (*SD = 8.41, Min_*age*_* = 18, *Max*_*age*_ = 68), and approximately 56.1 percent of participants were male (166 male, 130 female) coming from a diverse set of ethnic backgrounds.

#### Methods and Measures

We adapted the vignette used in Tiedens et al. (2000) to create three different versions that account for intense, moderate, and no anger expression intensity levels (see Appendix A). The first half of the revised versions that explain the anger-provoking scenario between leader X and assistant Y was almost identical to the original vignette by Tiedens et al. (2000), while the latter half that describes leader X’s behaviors was revised to include variation in anger expression intensity displayed by the leader. Each participant received a vignette with intense, moderate, or no anger expression, which according to Study 1 results, integrated emotional expression categories for generic, facial, vocal, and physical expression of anger. In the no anger vignette version, neutral words – calm, look, speak, and walk—were integrated to express neutrally tempered emotion.

To examine the effect of leader gender, the leader was addressed as either Mr. X or Ms. X. As a result, six vignettes – 2 (gender: male vs. female leader) × 3 (intense, moderate, and no anger) – were created and randomly assigned to participants. In other words, each vignette described either a male or female leader showing intense, moderate, or neutral levels of anger expression.

Perceived leadership effectiveness was measured by four items developed by Hais et al. (1997), asking to what extent X has qualities of good leadership, if these qualities match participant’s image of a good leader, if X behaved as a leader should, and if X would be an effective leader. Participants rated leader X on a 7-point scale with 1 indicating “not at all” and 7 indicating “very much.” Since the results for the questions measuring leadership effectiveness were highly correlated (Cronbach α = 0.95), we averaged all four item scores and calculated an average composite score which we used in data analysis (see Appendix B).

Status conferral items were the same as in Tiedens (2001). Among five items, one item asked participants to guess the leader’s current hierarchical status and the other four items asked participants to evaluate the future status, power, independence, and rank of the leader based on an 11-point scale (1 = none, 11 = a great deal). The four items measuring the extent to which participants confer future status to the leader in the vignette showed good scale reliability (Cronbach α = 0.96) and were averaged and used for analysis.^1^

Lastly, we included the question “how strong do you think Mr. (Ms.) X showed his (her) anger?” 1 = not at all, 7 = extremely for manipulation check and collected demographic information such as gender, age, and ethnicity for further analysis.

#### Results and Discussion

##### Manipulation check

One-way ANOVA analysis on manipulation check item revealed that there were significant condition differences in participant perceptions of leader anger expression intensity, *F*_(2, 29__3__)_ = 27.45, *p* < 0.001. Planned contrast analysis showed that participants in the no anger expression condition (*M* = 4.06, *SD* = 1.92) perceived the intensity of anger expressed by the leader significantly lower than those in the intense anger expression condition [*M* = 5.6, *SD* = 1.38, *t*_(19__4.20__)_^2^ = 6.66, *p* < 0.001] and moderate anger expression condition [*M* = 5.32, S*D* = 1.35, *t*_(19__0.22__)_ = 5.35, *p* < 0.001]. Although participants in intense anger condition reported higher level of intensity in the anger expressed by the leader than those in moderate anger condition, the difference was not statistically significant.

##### Leadership effectiveness

To verify our hypothesis 1, 3, and 5, we conducted a 3 (intensity: intense, moderate, no anger) × 2 (leader gender: male vs. female) × 2 (observer gender: male vs. female) three-way ANOVA analysis with perceived leadership effectiveness. As summarized in Table 2, the overall ANOVA results reported significant main effects of anger intensity and observer gender.

**TABLE 2:** Three-way analysis of variance results.

Source of variance	DV: leadership		DV: future status	
	effectiveness		conferral	
	*F*	Partial η^2^	*F*	Partial η^2^
Anger expression intensity (A)	14.10***	0.090	3.66*	0.025
Leader gender (B)	0.48	0.002	0.23	0.001
Participant gender (C)	17.77***	0.059	14.20***	0.048
A * B	1.02	0.007	0.18	0.001
A * C	0.24	0.002	0.17	0.001
B * C	0.01	0.000	0.01	0.000
A * B * C	0.25	0.002	1.38	0.010

*p < 0.05, ***p < 0.001.

First, the significant main effect of anger expression intensity indicates that there are significant differences in the perception of *leadership effectiveness* between three intensity conditions, *F*_(2, 2__84__)_ = 14.10, *p* < 0.001, partial η^2^ = 0.09, which supported our first hypothesis that anger intensity will have a significant effect on leadership perception. *Post hoc* comparison (Duncan’s test, *p* < 0.05) revealed the difference lies between the no anger expression condition (*M* = 4.92, *SD* = 1.44) and the other two conditions, moderate anger expression condition (*M* = 3.55, S*D* = 1.94) and intense anger expression condition (*M* = 3.89, *SD* = 2.01). Given that there was no significant difference between moderate and intense anger expression condition, our results demonstrate that in an anger-provoking situation, a leader who successfully controls emotional expression is perceived as a more effective leader than a leader who expresses anger regardless of the intensity of their anger expression.

Next, in analyzing observer gender, we found a significant main effect of observer gender on leadership effectiveness perception, *F*_(1__, 284__)_ = 17.77, *p* < 0.001, partial η^2^ = 0.059. Regardless of the leader gender, male participants (*M* = 4.58, *SD* = 1.81) rated the leader significantly higher in their leadership effectiveness than female participants did [*M* = 3.63, *SD* = 1.87, *t*_(273.04)_ = 4.39, *p* < 0.001].

Contrary to expectations, leader gender did not have any significant effect on leadership effectiveness *F*_(1, 284)_ = 0.48, *p* = 0.488, and the two-way interaction effect between anger intensity and leader gender was not significant, *F*_(2, 284)_ = 1.02, *p* = 0.361. In other words, participant evaluation of leadership effectiveness in response to leader display of anger did not change as a function of the leader gender. Regardless of the leader gender, the general tendency to rate a leader showing no anger as more effective remained the same.

Lastly, the three-way interaction effect by anger intensity, leader gender, and observer gender was not statistically significant, *F*_(2, 284)_ = 0.25, *p* = 0.781, which means our hypothesis 5 was not supported. While observer gender had a direct effect on leadership perception, it did not interact with anger intensity and leader gender in a meaningful way.

##### Future status conferral

Future status conferral To test our remaining hypotheses 2, 4, and 6, we conducted a 3 (intensity: intense, moderate, no anger) × 2 (leader gender: male vs. female) × 2 (observer gender: male vs. female) three-way ANOVA analysis with future status conferral. Similar to our previous analysis with leadership effectiveness, we found significant main effects of anger intensity and observer gender (Table 2).

First, there was a significant difference in future status conferred to the leader in the vignette between intense, moderate and no anger conditions, *F*_(2, 2__84__)_ = 3.66, *p* < 0.05, partial η^2^ = 0.025, which supports our second hypothesis. When we conducted *post hoc* comparison analyses (Duncan’s test, *p* < 0.05), we found a significant difference only between no anger expression condition (*M* = 7.72, *SD* = 2.08, *p* < 0.05) and moderate anger expression condition (*M* = 6.66, *SD* = 2.55). In contrary to the result with leadership effectiveness, the no anger expression condition did not significantly differ from intense anger expression condition (*M* = 7.03, *SD* = 2.80).

Similarly, in the case of *future status conferral*, there was a significant difference between the male and female participants, *F*_(1, 284)_ = 14.20, *p* < 0.001, partial η^2^ = 0.048. Again, male participants (*M* = 7.68, *SD* = 2.41) granted higher status to the leader than female participant [*M* = 6.52, *SD* = 2.50, *t*_(272.36)_ = 4.02, *p* < 0.001]. This reveals that male observers were significantly more positive when evaluating a leader in terms of future status as well as leadership effectiveness than female observers. Stated in reverse, female participants were overall more critical when rating leaders regardless of the leader gender and anger expressed.

As shown in Table 2, neither the two-way interaction effect of leader gender and anger expression intensity, *F*_(2, 284)_ = 0.175, *p* = 0.839, nor the three-way interaction effect by all three variables, *F*_(2, 284)_ = 1.38, *p* = 0.253, were statistically significant. This means leader gender and observer gender did not affect the relationship between anger expression intensity and future status conferral and hypothesis 4 and 6 were not supported.

##### Additional observations

In spite of insignificant interaction effects, examining descriptive statistics data revealed some insightful observations. In Table 3, the means and standard deviations for each condition created by the combination of anger expression intensity level, leader gender, and observer gender are presented.

**TABLE 3:** Means and standard deviations by anger intensity, leader gender, and observer gender conditions.

		Leadership effectiveness				Future status conferral			
Anger intensity	Leader gender	Observer gender		Leader gender Total	Anger intensity Total	Observer gender		Leader gender Total	Anger intensity Total
		Male	Female			Male	Female		
Intense	Male	4.28 (2.10)	3.43 (2.06)	3.90 (2.10)	3.89 (2.01)	7.64 (2.79)	6.38 (2.85)	7.08 (2.86)	7.03 (2.80)
	Female	4.14 (1.97)	3.48 (1.89)	3.87 (1.95)		7.17 (2.85)	6.69 (2.72)	6.98 (2.78)	
Moderate	Male	4.02 (1.80)	3.15 (2.14)	3.47 (2.05)	3.55 (1.94)	6.83 (2.51)	6.23 (2.64)	6.45 (2.58)	6.66 (2.55)
	Female	4.17 (1.92)	2.83 (1.46)	3.63 (1.85)		7.67 (2.43)	5.71 (2.24)	6.87 (2.53)	
No	Male	5.01 (1.62)	4.11 (1.45)	4.72 (1.61)	4.92 (1.44)	8.17 (1.93)	6.64 (1.97)	7.68 (2.06)	7.72 (2.08)
	Female	5.50 (0.86)	4.75 (1.39)	5.15 (1.19)		8.14 (1.90)	7.34 (2.33)	7.76 (2.13)	

**Participant gender Total**		4.58 (1.81)	3.63 (1.87)			7.68 (2.41)	6.52 (2.50)		

*The values in parenthesis indicate standard deviations.

To explore any potential effect of leader gender that might not have been captured in statistical analysis, we compared the mean values by leader gender. The most interesting observation is that for both leadership effectiveness and status conferral, the highest ratings were given to a calm female leader [leadership: *M* = 5.15, *SD* = 1.19; status: *M* = 7.76, *SD* = 2.13] and the lowest ratings were given to a male leader showing moderate anger (leadership: *M* = 3.47, *SD* = 2.05; status: *M* = 6.45, *SD* = 2.58).

Next, to examine observer gender together with leader gender, we arranged the twelve scores for each dependent variable in rank order. In addition to the overall main effect of observer gender, examining the ranking by mean scores reveals an interesting observation (see Table 3). Although female observers tended to give lower ratings than male observers did, when they evaluate a female leader expressing no anger, their rating scores were located within the top half out of twelve for both leadership effectiveness (3rd) and future status conferral (5th). Similarly, for both dependent variables, the only male ranking within the bottom half was a male leader expressing moderate anger (leadership: 7th, status: 8th).

## Discussion

### Conclusion

This study explores how anger expression of a leader affects individual perception of leadership effectiveness and future status conferral. In an attempt to discern reasons behind conflicting findings on the effect of leader anger display, we examined the intensity of anger display, the gender of the leader, and the gender of the observer as three key dimension variables that may affect perceptions of leadership and status. In Study 1, we ran a pretest to select terms that appropriately indicate intense and moderate levels of anger expression for generic, facial, vocal, and physical expression categories. In Study 2, the selected emotional terms were used to manipulate anger expression intensity of a leader in a hypothetical scenario and attempted to isolate and quantify the effects of our independent variables – anger expressed intense, moderate, or not at all and gender of leader and observer. Our undertaking to explore dimensions that affect leadership effectiveness and status conferral unveiled some interesting and significant findings.

First, there was a strong preference for a calm leader to an angry leader. Study participants perceived the angry leader who expressed either moderate or intense anger as less effective in leadership when compared to a leader who showed calmness in what may otherwise be interpreted as an anger-provoking situation caused by external circumstances. Similarly, in the case of future status conferral, a calm leader was conferred higher status than a leader showing moderate anger.

Next, while no anger expression was more positively related to both leadership effectiveness perception and future status conferral than anger expression, we found different patterns in relation to moderate and intense anger expression depending on the dependent variable. When leadership effectiveness was rated, no anger display was more beneficial than showing moderate and intense anger. In contrast, when future status was evaluated, display of no anger significantly differed only from moderate anger expression. In other words, in contrast to leadership effectiveness, for status conferral a leader showing intense anger was not conferred significantly lower future status than a neutrally tempered leader.

Third, contrary to our expectations, there was no significant main effect or interaction effect of leader gender. Namely, the effect of anger expression did not differ by gender of the leader. Even though we expected that female leaders would experience more negative evaluations when displaying their anger in public due to the inconsistency between gender norms and managerial expectations, we did not find any evidence of unfair treatments against the female leader in particular. Rather, an examination of mean values demonstrated that our study participants rated a female leader showing no anger to be the most effective in leadership and the highest in future status and a male leader showing moderate anger to be the least effective and the lowest.

Lastly, we found a significant main effect of observer gender such that male participants rated leaders, regardless of their gender, more effective in leadership and higher in future status than female participants. In other words, female participants were significantly more critical than their male counterparts. A separate analysis of mean evaluations for both leadership effectiveness and status conferral unveiled a telling and insightful observation – that of the twelve scores by anger intensity and leader gender, evaluations by female observers accounted for five out of six of the lowest scores with only women expressing no anger ranking in the top quartile for leadership effectiveness. This trend was even more pronounced for status conferral with female participant rankings comprising the bottom five average ratings and with no rankings by female participants making it into the top quartile. When reviewing the mean values in rank order, again we found that a calm female leader and a male leader displaying moderate anger stood out beyond the main effect of observer gender.

### General Discussion

Consistent with previous studies evaluating the effects of anger expression on perception of leadership effectiveness (Lewis, 2000; Sinaceur and Tiedens, 2006), in our study participants evaluated the leader who did not outwardly show anger as more effective in leadership than a leader who displayed moderate or intense anger. In contrast, our findings with future status conferral provide somewhat different implications from what prior research suggested (Tiedens, 2001; Brescoll and Uhlmann, 2008). Earlier studies reported that angry people were perceived higher in status than sad people were. In our study, when anger display was compared with no anger display, no anger was associated with higher future status than anger expression. Taken together, we can conclude that while showing anger may confer higher status than showing sadness, controlling anger is more helpful in status conferral than showing anger. This finding highlights the importance of selecting a comparison state in understanding the effect of anger expression correctly. Based on prior research, we might have developed a positive view of anger in terms of status conferral; our research refutes that perspective by showing that controlling anger is more instrumental in acquiring perception of higher future status.

Next, in our analysis of anger expression intensity, we found that anger expression can exert differential effects on individual perception and evaluation of leadership and status conferral according to its intensity. An overall insightful finding is that for both leadership effectiveness and status conferral, participants in our study gave lower evaluations for leaders who express moderate anger than a leader who expresses no anger. This is inconsistent with what is predicted by the Dual-Threshold model of Anger by Geddes and Callister (2007). This model proposed that expressed anger located in the middle between deviant anger and suppressed anger will yield more positive outcomes in groups and organizations. In our research, showing moderate level of anger was less beneficial than staying neutral in observer perception of the expressor’s leadership and status. This inconsistency might stem from which outcome is being investigated. For instance, expressing moderate anger can facilitate group processes by sending a warning signal of inadequate performance but possibly have a negative impact on how the expressor is perceived and evaluated.

Furthermore, these findings indicate that moderate anger expression as the leader’s response to the given situation can be viewed as a weakness that makes them appear less effective in leadership skills and less eligible for higher status. While in the case of leadership effectiveness the significant difference was found between expression of anger and no anger regardless of anger expression intensity, when we looked at future status conferral as a dependent variable, the significant difference was observed only between moderate and no anger expression. Aligned with research on gender and anger, we infer that such may be related to observer internal attribution of the leader behavior affecting the leader’s ability to self-regulate and control their emotions. The fact that there was no significant difference between intense anger and no anger conditions in future status conferral reveals the importance of a critical dimension – how anger intensity affects leadership effectiveness and status conferral differently. Based on this finding, we believe that intensity effect should be considered in future studies on anger expression. Emotional expression in general and anger expression in particular are not binary, and hence, future studies should incorporate distinctive features associated with different levels of intensity. Thus, researchers should be cognizant of the effect of intensity and try to avoid generalizing a finding at a certain level of anger expression to the entire spectrum of anger expression.

The third valuable learning from this study was that the gender of the leader expressing the emotion did not significantly affect the evaluation of leadership effectiveness and status conferral. However, we found a surprising degree of consistency in descriptive data for both male and female leaders. One insightful finding is that for both leadership effectiveness and future status, when the female leader expressed no anger, she received the highest evaluations. Equally insightful was the finding that the lowest evaluations were given to the male leader expressing moderate anger. We believe this may be partially reflective of the changing view on the leadership in general and the role of female leaders at work. Perspectives on good leadership have changed in regard to contemporary organizational cultures that embrace social and technological change (Avoilio, 1999), and this change has been strengthened with the increase in the number of female leaders at the workplace. Results are also consistent with Eagly and Johnson’s (1990) meta-analysis showing that organizational culture tends to socialize managers to minimize stereotypical gender behavior and that managers are less likely to be perceived in terms of their gender role. Research finds that male leaders are rewarded for demonstrating more communal or non-confrontational leadership styles (Eagly et al., 1992; Pratch, 1996). Observing and experiencing more female leaders in organizations was found to change people’s perception of leader roles (Dasgupta and Asgari, 2004; Beaman et al., 2009). The changing social trend and the ensuing reduced gap between gender norms and leadership qualities might explain why angry female leaders in our study did not receive punitive evaluations.

Lastly, related to the role of observer gender, we found that male observers were significantly more positive in their perceptions of leadership and future status conferral or – oppositely – that female observers were considerably more critical in evaluating leaders than their male counterparts. This finding is perhaps one of the most insightful, as while there is consistent reporting on informal barriers that collectively contribute to the glass ceiling, aside from gender pay gap, identification of factors might contribute to bias and can serve as an explanation for why female leaders may receive lower evaluations or encounter barriers in terms of assessment, promotion, and compensation. Social dominance theory (Sidanius et al., 2004) seeks to explain social group oppression by identifying specific individual and structural factors that lead to group-level bias. Often, power legitimizes hierarchal structures that may contain male bias and reward traditionally masculine leadership characteristics. As an in-group, male observers may perceive human resource management policies to be much fairer than their female counterparts perceived. In other words, male participants are more positive as they are part of the power in-group and perceived policies related to gender discrimination in recruitment, assessment, promotion, and compensation, as far less discriminatory than female leaders, regardless of their level (Ashihara et al., 2019). Likewise, women who aspire to leadership roles perceive the existence of barriers to entering these roles and advancing to higher levels within an organization and those perceptions may differ from their male counterparts. As women enter male-dominated leadership roles where autocratic styles are common, they may encounter significant bias or feel challenged to act differently than their gender stereotype and culture may have embedded, providing additional challenge as it may not be their natural tendency. One interesting conclusion is that women may in fact be holding themselves back as their own views may construct barriers that prevent them from leaning in rather than opting out.

### Limitations and Future Direction

As with other experimental studies, this study is also vulnerable to the criticism that it lacks external validity. In Study 1, our sample primarily consists of young college students. Lab experiments with student samples using artificial materials like vignettes may arouse the concern over their generalizability to the real world. Furthermore, the use of undergraduate students and their relatively lower status may alter their perception as they may have a tendency to empathize with the anger recipient rather than the leader expressing anger in the vignette. To reduce that demographical bias, we recruited a diverse sample from Amazon MTurk, which comprised roughly two thirds of Study 2 participants. We recognize that the discrepancy in sample demographics between Study 1 (mostly students) and Study 2 (students and general people) might have altered results and the possibility is that the emotion terms chosen by students in Study 1 could have been interpreted differently by people in the MTurk sample. While we did compare results to other studies on anger terms (Shaver et al., 1987), we nonetheless acknowledge that terms are intrinsically subject to interpretation and embedded in cultural and generational use. Future research should be conducted with a more diverse and greater number of participants that are diverse in terms of age, work experience, and social status to enhance external validity of study findings and consistency in sample characteristics between studies.

We also recognize the inherent limitations of a survey method to convey genuine anger with nuances or in a context that allows participants to evaluate the appropriateness of the anger. Verbal and non-verbal cues are important when conveying the true meaning of the message (Galin et al., 2007). Argyle and colleagues support the research finding that non-verbal cues are more salient than verbal cues (Argyle et al., 1971). Even though our research included terms describing non-verbal expression of anger, its effect might be different from the manipulation method presenting non-verbal cues via more vivid media such as video. One reason why our intensity manipulation between moderate and intense anger was not successful might be related to the limitation embedded in describing emotions using only words. We also recognize that terms selected for intense and moderate anger are subject to interpretation and that the specific terms for moderate anger “snap at,” “frown,” and “stalk” may be more indicative of internal or personality traits for anger expression. Future research would need to consider other manipulation techniques which can deliver non-verbal cues to the subjects more directly.

Given that this study focuses on the interpersonal perception between a leader and subordinates, we suggest that a study considering real organizations and observations of real leader–subordinate interactions would enhance the ecological validity of these study results. Compared to the experimental situation where the subjects are asked to regard a person whom they have never met before as their leader, real organizational contexts have a history of relationship among organizational members including leaders and subordinates. As such, other variables might come into play when subordinates observe their leader’s anger display in real situations. More contextual information about the situation itself, the leader’s emotional characteristics, and actual expression of anger including responses of other colleagues would likely influence the perception of leadership effectiveness. Thus, field studies using a variety of methods such as survey, interview, and observation can be considered as a next step for future direction. The replication of similar results in a more natural setting which integrates non-verbal cues and a more realistic leader–subordinate relationship that considers gender and differing anger conditions would be important in ascertaining the validity of anger perception and leadership status conferral.

We also propose more in-depth studies looking at the gender of the participant and how male and female observers differ in their response to leadership and to explore nuances of how their views differ for other independent variables. It may be worthwhile to understand more dimensions on the leadership effectiveness for female leaders as expressing no anger is in line with being unemotional and gender incongruent. We should seek to identify leadership strategies that enable professional women to express their power and position in challenging situations without incurring penalty. In line with this, it may be worth exploring how ratings of effectiveness differ when observers are provided with direction for source of the emotion display as having internal or external attribution.

Furthermore, we hope future studies will expand the scope of our research by examining different contexts for anger display and various outcome variables in response to the leader’s anger expression. For example, we believe that domain-specific anger expression introduced by Bongard and Al’Absi (2003) can offer valuable insights about emotion display at the workplace. It was found that people show their anger more openly and strive to control anger less in more private settings while the opposite pattern was observed in public settings. In particular, men and women showed different anger expression styles in different settings. Women reported higher anger control and less outward anger expression than did men at the workplace. If we could manipulate the contextual settings for anger display (e.g., group vs. private meeting), we might be able to learn more sophisticated approach men and women take in terms of emotion regulation at work.

In this paper, we primarily focused on how a leader’s display of anger affects employee perception of a leader’s effectiveness and future status conferral by looking at gender of leader and anger expression intensity. Leader’s emotional display can also impact employee behavioral intentions or actual behavior as well as their cognitions or emotions. An interesting segue is whether leaders who are perceived as expressing negative emotions influence the work patterns and corporate culture in a negative way. For instance, would working with an anger-prone leader increase obsessive tendencies (workaholism) which may impact work–family balance? (Mazzetti et al., 2019). Given the influential power of a leader on followers, understanding how a leader’s emotion display influences a manifold of employee behavior would be another important task.

While our findings revealed some value in including intensity as a dimension of anger expression and demonstrated how the intensity factor may influence leadership effectiveness and conferral differently, we also found that even if women “lean in” (Sandberg, 2013), the standards they hold themselves to may in fact provide barriers to their own success. Our findings on leader gender support the views of Melinda Gates who was quoted as saying “a woman with a voice is, by definition, a strong woman” and of Madeline Albright who said women leaders expressing no emotion are seen as more rational. While female leaders are perceived with some degree of gender parity, the fact that female observers hold more critical views of leaders than their male counterparts might lead aspiring women to perceive the existence of barriers and provide obstacles to their own advancement.

## Data Availability Statement

The datasets generated for this study are available on request to the corresponding author.

## Ethics Statement

The studies involving college affiliates were reviewed and approved by the UCLA IRB. Participants recruited via Amazon Mturk read the study description and provided their consent online. Participants were also informed of their ability to withdraw from the study should they for some reason decide to do so.

## Author Contributions

DY was responsible for idea development, data collection and analysis, literature review, and writing. HJ contrived the research idea and study design, took part in writing and editing, and advised the overall process. KA worked on theoretical framework building, interpretation of research findings, literature review, writing, and editing. All authors contributed to the preparation of the manuscript and approved the final version of the manuscript.

## Conflict of Interest

The authors declare that the research was conducted in the absence of any commercial or financial relationships that could be construed as a potential conflict of interest.

## Footnotes

1 For both leadership and status measure, we used the same original format as used in prior studies (Hais et al., 1997; Tiedens et al., 2000), which explains why each scale employed different Likert scales (7-point vs. 11-point).

2 Levene’s tests for equality of variance were significant, which means the equality of variance assumptions are not met. Thus, we report *t*-test statistics and df offered in the case of “equal variance not assumed.” Please note that the main findings remained the same regardless of whether this equality of variance assumption is met or not.

## Appendix A

### Vignettes for Study 2

“Mr./Ms. X and his/her assistant Y had a meeting with a client to present an idea for advertising a new product. Y had the materials for the slide presentation in the car and followed Mr./Ms. X, who had the directions, to the meeting. Mr./Ms. X and Y lost each other in traffic. Mr./Ms. X got to the meeting on time but could not do the presentation because the visual aids were in Y’s car. By the time Y got there, the client was furious and they lost the account. *When they were walking out of the client’s office, Mr./Ms. X looked* ***furious/annoyed/calm*** *(general). Mr./Ms. X* ***glared at/frowned at/looked at*** *(facial) Y, and****yelled at/snapped at/spoke to*** *(vocal) Y, “why were you late?” When Y is about to make an excuse, Mr./Ms. X said, “let’s go back to the office” and* ***stomped/stalked/walked*** *(physical) out of the building.*

^∗^ This is a modified version of the vignette used by Tiedens et al. (2000). The part in italic has been newly added for intensity manipulation and the emotional terms are presented in the order of intense/moderate/neutral condition.

^∗^ () is not shown to study participants.

## Appendix B

### Measures for Study 2

Leadership effectiveness: 7-point Likert scale (1 = “not at all,” 7 = “very much”).

• To what extent does Mr./Ms. X have qualities for good leadership?
• To what extent does Mr./Ms. X match your image of a good leader?
• To what extent did Mr./Ms. X behave as a leader should?
• To what extent would Mr./Ms. X be an effective leader?

Status conferral: 11-point Likert Scale (1 = “none,” 11 = “a great deal”).

• How much status does Mr./Ms. X deserve in his/her future job?
• How much power does Mr./Ms. X deserve in his/her future job?
• How much independence does Mr./Ms. X deserve in his/her future job?
• How much rank does Mr./Ms. X deserve in his/her future job?
